# Evaluation of Polycyclic Aromatic Hydrocarbons (PAHs) in Pork Meat Cooked with Two Different Methods

**DOI:** 10.3390/molecules30091886

**Published:** 2025-04-23

**Authors:** Chiara Conchione, Silvia Socal, Laura Barp, Sabrina Moret

**Affiliations:** Department of Agri-Food, Environmental and Animal Sciences, University of Udine, 33100 Udine, Italy; chiara.conchione@uniud.it (C.C.); laura.barp@uniud.it (L.B.)

**Keywords:** polycyclic aromatic hydrocarbons (PAHs), microwave-assisted saponification (MAS), ultra-high-performance liquid chromatography with spectrofluorometric detection (UHPLC-FLD), grilled meat, cooking methods

## Abstract

During domestic grilling, polycyclic aromatic hydrocarbons (PAHs), which include genotoxic and carcinogenic compounds, can be produced as a result of fat pyrolysis, leakage of cellular juices onto the heat source, and incomplete combustion of fuel. This study aimed to assess the formation of PAHs in pork neck cooked using two different grilling methods (traditional flat grill with beech charcoal and asado grill with beech wood flame) under controlled conditions, with cooking stopping at a core temperature of 72 °C. The impact of marinating and cooking speed (fast or slow) was also evaluated over three cooking sessions. After grilling, the meat samples underwent microwave-assisted extraction, purification through solid-phase extraction (SPE), and analysis using ultra-high-performance liquid chromatography (UHPLC) with spectrofluorometric detection. Statistical analysis was performed using ANOVA (R software, version 4.3.0). None of the samples exceeded the legal limits for benzo[a]pyrene (BaP) and PAH4 (sum of chrysene, benzo[a]anthracene, BaP, and benzo[b]fluoranthene). However, the asado grill showed a significantly higher average PAH contamination (1.21 µg/kg of BaP and 3.92 µg/kg of PAH4) compared with the traditional grill (0.22 µg/kg of BaP and 1.71 µg/kg of PAH4). Marinating and cooking speed did not have a significant impact on PAH levels.

## 1. Introduction

Pork meat is one of the most popular meats in the world, particularly in Central Europe and East and Southeast Asia. Nowadays, cooking meat has become a real cult, in which techniques and preparations are aimed at obtaining the best organoleptic result while minimizing the possible formation of hazardous contaminants. Grilling meat gives the food highly valued organoleptic characteristics. However, if not conducted properly (high temperature for a long time, improper selection of fuel, fat dripping on burning embers, etc.), it can lead to the production of genotoxic and carcinogenic polycyclic aromatic hydrocarbons (PAHs) that expose consumers to an increased risk of developing certain types of cancer [1,2,3,4,5].

PAHs are a class of environmental and food processing contaminants with 2–6 fused benzene rings that originate from the incomplete combustion of organic matter at high temperatures (500–700 °C) from both natural and anthropogenic sources [6]. Compounds with 4–6 benzene rings, known as “heavy PAHs”, are classified as genotoxic and carcinogenic substances [7]. According to Marques et al. (2022) [8], there is a positive and statistically significant correlation between PAH dietary intake and the risk of mortality from lung or tracheal cancer.

The exact mechanism of PAH formation in grilled foods is not precisely known, but it has been demonstrated that PAHs can form from the pyrolysis of fat and/or smoke produced by incomplete combustion of charcoal or open fires [9]. In addition to the equipment used and its geometry, many processing parameters, such as the type of fuel, cooking temperature and duration, type of meat (especially its fat content), and pretreatment can significantly influence the PAH content of grilled meat [5,9]. As recently reviewed by Duedahl-Olesen and Ionas (2022) [10], several authors have studied different charcoal-cooking techniques, demonstrating that maintaining stable combustion after the burning charcoal flames have subsided can help reduce the PAH content. In addition, removing the dripping fat and smoke generated by the charcoal has been shown to decrease the formation of PAHs.

Alongside equipment for traditional cooking on a flat grill, equipment to simulate traditional Argentine grilling (asado) on a smaller scale is becoming popular in the Italian market. Traditional asado cooking comes from South American culture, specifically from gauchos in remote areas of the Argentine pampas, and involves skewering large pieces of meat on sticks, strictly green, to prevent them from burning during cooking. Owing to special horizontal supports, the meat is suspended at an angle near the burning coals, giving it the classic “crucifixion” (a la cruz) appearance. It is eaten little by little, cutting off the superficial cooked part. This cooking method utilizes the heat given off by flames or embers, and the meat is cooked slowly by radiation (indirect cooking). The food is exposed to a lower temperature than in classic grilling (direct cooking) but for a longer time.

While previous research has shown that direct flame exposure and fat drippings contribute significantly to benzo[a]pyrene (BaP) formation, most recent studies have focused on charcoal, infrared-ray, electric grills, or grill pans. The findings that infrared-ray, electric, and pan grilling prevent BaP formation align with the general understanding that avoiding direct combustion exposure reduces contamination [4,9,11]. However, to the best of our knowledge, there is a lack of research on PAH formation using asado grills available on the market for domestic use. Therefore, the main purpose of this study was to assess the production of PAHs in pork cooked with a commercial asado grill. The grill used was equipped with a brazier for wood fire, an adjustable-tilt metal frame with skewers for cooking meat over the flame, a grease drainage system to prevent drips, and a metal cover to maintain and radiate heat. The study also examined the impact of marinating the meat before cooking and two different time–temperature combinations to reach the internal temperature of 72 °C more or less quickly. The results of three cooking sessions on the asado grill were compared with those of cooking conducted in parallel on the same pork neck meat on a traditional flat grill under similar conditions, but using charcoal from the same beech wood (prepared in a separate brazier).

## 2. Results and Discussion

### 2.1. Samples and Cooking Conditions

Selecting pork neck, a high-fat cut, was intentional in order to investigate PAH formation during grilling. Fat drippings play a crucial role in the generation of PAHs, as they fall onto the heat source, leading to pyrolysis, the thermal decomposition of organic matter in the absence of oxygen [12]. This process produces volatile organic compounds that, when incompletely combusted, result in the formation of PAHs, including known carcinogens such as BaP. Moreover, high-fat meats release more lipids during grilling, which increases the likelihood of flare-ups and smoke deposition on the meat’s surface. These factors are major contributors to the generation of PAHs [10].

The formation of PAHs during wood combustion is significantly influenced by the type of wood used, as each species has a unique chemical composition with variations in lignin, cellulose, hemicellulose, and extractives. Beech wood was chosen for grilling primarily due to its local availability and widespread domestic use. However, several studies suggest that its combustion may produce higher PAH levels compared with other wood types [13,14], likely due to its high lignin content and the presence of extractive compounds that contribute to PAH formation.

Along with the type of meat and wood used for grilling, the core temperature of the product was kept constant throughout the experiment. Specifically, the cooking process was carried out until the meat reached a core temperature of 72 °C, ensuring food safety by eliminating the risk of trichinellosis, a zoonotic disease caused by parasitic nematodes [15].

Table 1 displays data on the heating source temperatures, cooking durations, and climatic conditions from three experimental sessions. The stability of temperature and cooking duration was significantly impacted by climatic conditions and cooking methods. Warmer weather resulted in shorter cooking times, while colder and more humid conditions extended them, making temperature control more challenging. In the asado setup, heat intensity was regulated by adjusting both the quantity of burning wood and the distance between the flame and the meat, with temperatures ranging from 350 °C to 540 °C. In the traditional flat grill, temperature control was achieved by varying the amount of charcoal used, maintaining an average of 300 °C for slow cooking and around 500 °C for fast cooking.

### 2.2. Weight Loss and Fat Content After Cooking

Table 2 provides data on weight loss and fat content in the meat cooked using two different methods, flat grill and asado grill, under varying conditions (fast and slow cooking, with and without marinade). The values are expressed as average (*n* = 6) percentages, with standard deviation (SD) and relative standard deviation (RSD) reported.

The fat content in both cooking methods, ranging from 21.5% to 26.6%, was relatively consistent across the different cooking modes, with only slight variations.

The weight loss, ranging from 29.0% to 37.2% across the different cooking conditions, was induced by cooking, primarily due to water evaporation and fat dripping. Statistical analysis using a *t*-test (α = 0.05) revealed no significant differences among the different cooking methods, cooking speeds, and marinade treatments. Therefore, weight loss can be considered consistent across the various cooking scenarios, allowing for a direct comparison of the PAH content in the cooked meat without needing to account for variations in the meat’s weight before cooking.

The cooking conditions did not significantly influence the amount of fat in the meat, which remained relatively stable across all conditions (*t*-test, α = 0.05). This suggests that the impact of different grilling techniques on the residual fat content of the meat was negligible.

It is important to underline that, different from the flat grill, the asado grill was specifically designed to minimize the fat dripping onto the burning wood, featuring a dedicated channel aimed at collecting as much of the fat as possible during cooking (see Figures S1 and S2).

### 2.3. Extraction and Analytical Determination

During method optimization, various parameters were carefully evaluated to ensure the highest sensitivity and accuracy in determining PAHs.

One crucial aspect tested was the sample amount used for extraction. Different quantities were analyzed, namely 1, 2.5, and 5 g. While increasing the sample size generally improved sensitivity, it also presented practical issues. Using 5 g of meat resulted in a significant emulsion forming between the aqueous and organic phases after saponification, making phase separation more challenging. Additionally, some residual fat was observed, indicating incomplete saponification. Conversely, using only 1 g of sample, while avoiding emulsion problems, led to lower sensitivity. Ultimately, 2.5 g was identified as the ideal amount, striking a balance between sensitivity and complete saponification. The formation of emulsions was avoided by resting the sample overnight (about 12 h) before collecting the hexane phase.

Finally, chromatographic conditions were refined to optimize sensitivity and peak resolution. The method initially followed the conditions described by [16], with minor modifications made for improved performance. One key factor evaluated was the injection volume. Testing different volumes (5, 8, and 10 µL) showed that a volume of 8 µL was the optimal compromise, providing a balance between sensitivity and peak sharpness without causing peak broadening that could impact resolution.

**Figure 1:**
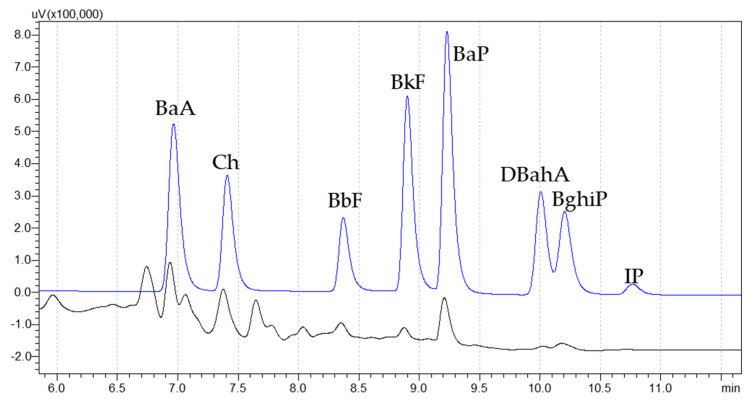
UHPLC-FLD chromatograms of PAHs standard at 2 µg/kg (blue trace) and an asado-cooked sample (black trace).

Figure 1 shows the UHPLC-FLD chromatograms obtained from the analysis of a standard mixture at 2 µg/kg of each heavy PAH, namely BaP, benz[a]anthracene (BaA), benzo[b]fluoranthene (BbF), chrysene (Ch), benzo[k]fluorathene (BkF), dibenzo[a,h]anthracene (DbahA), benzo[g,h,i]perylene (BghiP), and indeno [1,2,3-cd]pyrene (IP), and of an asado-cooked sample. The sum of these PAHs is referred to as PAH8.

### 2.4. Method Performances

Analytical performance parameters are summarized in Table S1. The method used to quantify PAH8 demonstrated excellent linearity across the tested concentration range of 0.1–10 µg/kg. The coefficient of determination (R^2^) values for all PAHs ranged from 0.9899 (BaP) to 0.9998 (BaA and BbF).

Recoveries and repeatability were evaluated using six replicates of meat samples spiked with a PAH amount equivalent to 2 µg/kg for each PAH8 (which aligns with the legal limit set for BaP in smoked meat). Average recoveries ranged from 79.5% for BaA to 98.1% for BaP, falling within the 50–120% range required by EC Regulation 836/2011 [17].

The RSD% of the six replicate analyses varied between 5.2% (BghiP) and 10.4% (IP).

The limit of detection (LOD) of the method in solvent (extrapolated from the lowest calibration point) was below 0.02 µg/kg for all PAH8, except for DBahA and IP (0.04 µg/kg). This ensures that the limit of quantification (LOQ) is well below the 0.9 µg/kg threshold in the presence of the matrix, as required by EU Regulation 836/2011 [17].

### 2.5. PAHs Related to the Different Cooking Conditions

Although UHPLC analysis is capable of detecting 2–3-ring light PAHs, the decision was made to focus the research exclusively on PAH8, which includes the most concerning and regulated heavy PAHs. In particular, special attention was given to PAH4 and PAH8, following the EFSA recommendations for PAH monitoring in food [7]. According to EFSA, PAH4 (BaP, BaA, BbF, and Ch) serves as the most reliable marker for PAH contamination in food, while PAH8 provides a more comprehensive assessment of total exposure to carcinogenic and genotoxic PAHs. The monitoring of these specific PAH groups aligns with European regulatory frameworks, which set maximum limits for PAH concentrations in various food products, including smoked meats [18].

**Figure 2:**
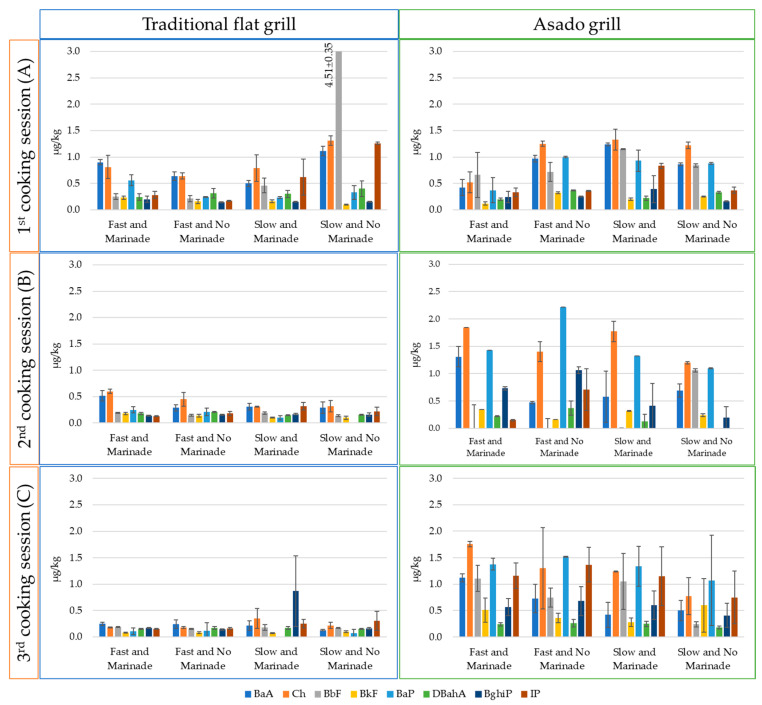
Average levels (µg/kg) of two replicates of each PAH8 in pork neck meat cooked in three different sessions under varying conditions using a traditional flat grill and an asado grill. “Fast” and “slow” refer to the cooking speed, while “marinade” or “no marinade” indicates whether the meat was marinated before cooking. Vertical bars represent standard deviations of two replicates.

Figure 2 presents the individual average PAH8 values (two replicates) detected in meat samples subjected to three different cooking sessions using both traditional flat grill and asado methods. The analysis also considers the influence of cooking speed (slow or fast) and the influence of marinating before cooking. The data are expressed as mean values (µg/kg) along with the corresponding standard deviation, based on two replicates. The complete data are provided in Table S2.

In the case of traditional grilling, the most prevalent PAHs were Ch and BaA, with average concentrations of 0.51 µg/kg and 0.45 µg/kg, respectively. Other heavy PAHs were present at lower concentrations, typically ranging between 0.13 µg/kg and 0.34 µg/kg. The highest PAH levels were observed during the first cooking session, while the contamination profile remained relatively consistent in the subsequent sessions. However, an exception was noted for IP and BbF, which reached significantly higher values of 1.26 µg/kg and 4.51 µg/kg, respectively, exclusively in the first session. Additionally, BghiP showed an elevated concentration of 0.87 µg/kg during the third session.

In the meat cooked using the asado, the most abundant compound was Ch, with an average concentration of 1.30 µg/kg, followed by BaP at 1.21 µg/kg and BbF and BaA at 0.84 and 0.78 µg/kg, respectively. The contamination levels across the three cooking sessions appeared to be fairly consistent. However, a greater variability in the data was observed in session C.

One of the most influencing factors on PAH production during grilling is the dripping fat, which undergoes pyrolysis and deposits PAH-laden smoke onto the meat’s surface [19]. Flat grilling, lacking a fat drainage system, exposes the meat to more PAHs generated by dripping fat onto hot charcoal. However, asado grilling produced higher levels of PAHs (see Figure 2). Other factors, such as the stage of combustion (it is known that the highest production of PAHs occurs during the early stages) and prolonged contact with the combustion fumes due to the design of the asado grill used (the metal cover visible in Figure S2) may explain these results. The increase in PAH concentration was observed across all PAHs, but it was particularly significant for BaP, which showed a six-fold increase in the asado-cooked samples compared with traditional grilling.

According to Han and colleagues [20], PAH emission is highly dependent on combustion temperature and varies between wood and coal combustion due to different emission mechanisms. In coal combustion, higher temperatures reduce PAH emission by breaking down their supramolecular structure. In contrast, wood combustion promotes PAH synthesis, likely due to different chemical reactions occurring at high temperatures. Moreover, PAH emission is influenced not only by their formation but also by elimination processes, such as oxidation and transformation into elemental carbon. Coal combustion tends to convert PAHs into elemental carbon, reducing overall PAH emission. The combustion process itself also affects PAH formation. In the early stage, where flames dominate and oxygen levels are lower, low-molecular-weight PAHs are formed. As combustion progresses and temperatures rise, high-molecular-weight PAHs are synthesized in greater quantities.

The dynamics of PAH formation during wood combustion are highly relevant to the asado cooking method, where meat is grilled over an open wood flame. Unlike traditional charcoal grilling, where coals have already undergone high-temperature combustion (reducing PAH emissions), the asado method involves active wood burning, which can significantly increase PAH formation due to the synthesis reactions occurring at high temperatures. Wood combustion promotes the synthesis of PAHs, especially during the early combustion stage, when flames are present and oxygen levels are lower. This could explain why higher levels of PAHs were detected in meat cooked with the asado compared with the traditional flat grill, where the fuel had already been transformed into charcoal, minimizing PAH emissions. In addition, prolonged contact with combustion fumes due to the presence of the metal cover may have further contributed to the higher PAH contamination observed in the asado-cooked meat.

Several ANOVA (α = 0.05) tests were conducted (R software, version 4.3.0) to evaluate the impact of different factors on the levels of specific PAHs in grilled meat. The analysis focused on four key variables: (i) cooking method (flat grill and asado grill); (ii) grill mode (fast and slow); (iii) marinade (with and without); (iv) cooking session (session-to-session variations). The results revealed that the cooking method had the most significant impact on PAH formation, with a highly significant effect (*p* < 0.001) across all PAHs. This confirms that the choice between traditional flat grilling and asado cooking plays a crucial role in determining the final PAH levels in the grilled meat. The effects of grilling mode, marinade, and cooking session were less consistent, with their significance varying depending on the specific PAH analyzed. Interaction terms did not show strong significance, suggesting that each factor tends to act independently rather than in combination with others.

Regarding the influence of grilling time and temperature, many authors have reported a significant increase in the concentration of PAHs in meat with the increase in these two parameters [21]. Chen and Chen (2001) [22] suggested that under drastic heating conditions, more lipid oxidation occurs and more degradation products can be formed, which, in turn, results in higher formation of 5–6 ring PAHs.

Unexpectedly, the different time–temperature combinations used in this work, when “fast” and “slow” cooking was performed to reach a core temperature of 72 °C, did not significantly affect the formation of PAHs.

According to some authors [23,24], to minimize PAH formation, the grilling temperature should be reduced with a concomitant longer cooking time, while according to others [25,26], it is better to reduce the grilling time and avoid overcooking. Given the contradictory results in the literature and the variability in experimental parameters, it is challenging to draw conclusions based on time and temperature alone [10].

Regarding the marinated samples, previous studies [27,28,29] suggest that appropriate selection of ingredients (such as wine and spices) can influence the physicochemical properties of the meat and can reduce the level of PAHs in the final product. However, the expected reduction was not observed. A possible explanation could be that the marinade used was not sufficiently acidic and contained whole pieces of garlic and rosemary, which may not have effectively interacted with the meat surface to limit PAH formation.

Regarding the possible influence of environmental conditions, since it depends on the interaction of several factors that are difficult to control (intrinsic variability), data obtained (in double) from three different cooking sessions were treated as six replicates of the same experimental condition (inter-day repeatability).

**Figure 3:**
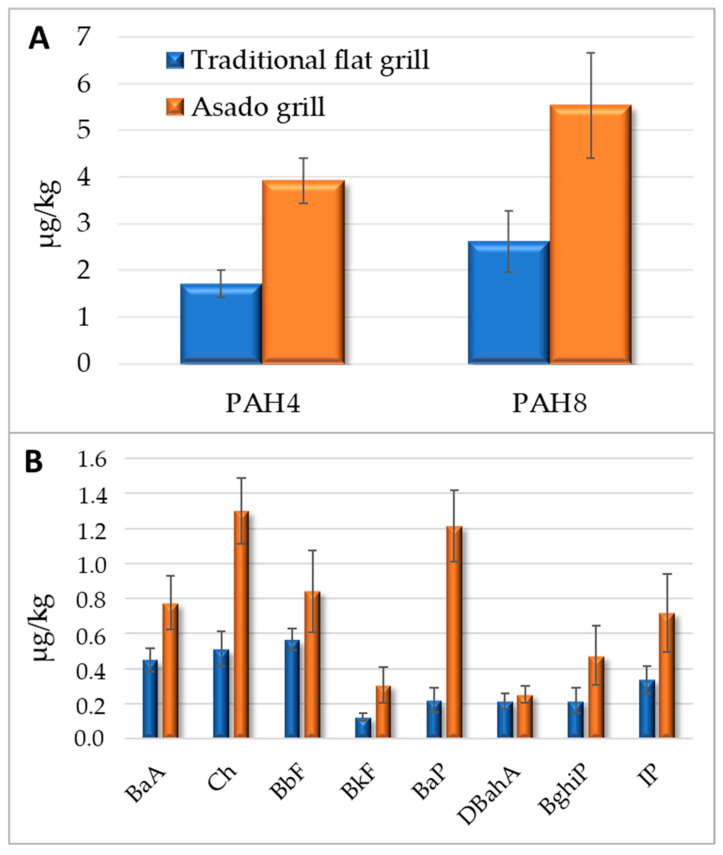
Average distribution of (**A**) PAH4 and PAH8 and (**B**) individual PAHs, expressed as µg/kg, in pork cooked using a traditional flat grill and an innovative asado grill. Vertical bars represent the standard deviations of 24 replicates.

Since, except for the cooking method, there were no significant statistical differences across the variables studied, the PAH4 and PAH8 levels were calculated based on the average of 24 replicates (3 cooking sessions with 2 replicates per variable: fast and slow cooking, marinated and non-marinated), and these are reported in Figure 3A, distinguishing between the two cooking methods: traditional flat grilling and asado grilling. Traditional grilling resulted in lower PAH4 and PAH8 formation, with an average value of 1.71 µg/kg and 2.62 µg/kg, respectively. These values were double in the meat cooked using the asado.

A strong positive correlation (R^2^ = 0.9001) between PAH4 and PAH8 levels was observed. As the concentration of PAH4 increases, the concentration of PAH8 also tends to increase. Furthermore, 90.0% of the variation in PAH8 levels can be explained by the variation in PAH4 levels, confirming the strong linear relationship between the two PAH groups. This suggests that PAH4 could serve as a good predictor for overall PAH8 contamination in grilled meat.

Levels of specific PAHs in food samples prepared using the two grilling methods are reported in Figure 3B. While in the case of the meat cooked with the traditional grill, the prevailing PAHs were Ch and BaA, in the case of the asado-cooked samples, the most abundant PAHs were, on average, Ch followed by BaP, BbF, and BaA.

The EFSA [7] sets specific limits for the presence of certain PAHs in food products, including smoked meat. Specifically, the legal limit for BaP is set at 2 µg/kg, and that for PAH4 at 12 µg/kg. No values above the legal limits were found, except for one sample obtained with asado (fast cooking with no marinade), which contained 2.2 µg/kg of BaP.

Focusing on the PAH levels found in the samples cooked using the traditional flat grill, comparisons can be made with data from the literature. Chung and collaborators [5] evaluated the effects of charcoal grilling, charcoal roasting, and gas roasting on PAH formation in beef and pork samples. They found that charcoal grilling produced the highest PAH levels in pork meat, with 2.90 µg/kg of BaP and 10.18 µg/kg of PAH8 (excluding BaA). However, the study did not provide information on wood type or the temperatures reached during cooking. These high PAH values were attributed to the pyrolysis of melted fat.

Rose et al. (2015) [30] found that pork chops grilled solely with charcoal had BaP levels of 1.90 µg/kg and 8.07 µg/kg of PAH4. Lee and colleagues [9] also investigated PAH formation during conventional grilling of pork meat, finding significant reductions (41–89%) in PAH4 levels when meat drippings and smoke were eliminated using an alternative grilling apparatus. They highlighted that the most important factor contributing to PAH production in meat grilling was the smoke resulting from the incomplete combustion of fat that dripped onto the fire. Their study reported BaP levels ranging from 1.14 µg/kg to 10.27 µg/kg and PAH4 levels ranging from 4.30 µg/kg to 33.17 µg/kg.

Our data revealed much lower PAH levels compared with the studies mentioned above, with BaP always below 0.6 µg/kg and PAH4 below 3 µg/kg. Probably, exposing the meat to hot coals (traditional grill) after the first combustion phase in which PAH production is higher and avoiding open flames prevented excessive temperatures that could trigger pyrolysis and PAH formation [11]. Regarding asado grilling, minimizing the dripping of fat probably helped to keep the amounts of PAHs generated low.

It is important to underline that the contamination levels found when cooking the pork under the controlled conditions reported, regardless of the cooking method, are of little concern. According to the EFSA opinion published in 2008 [7], the overall average dietary exposure to BaP and PAH4 across European countries for which data are available is 235 and 1168 ng per day, respectively. These contamination levels are associated with a low health concern for consumers [7]. Meat and meat products account for an average dietary exposure of 42 and 195 ng per day [7] for BaP and PAH4, respectively (about 18% of overall average dietary exposure). Assuming to consume asado-cooked pork twice per week (worst-case scenario), the resulting dietary exposure due to meat and meat products would be 46 and 147 ng per day for BaP and PAH4, respectively, which is not very different from the levels reported by EFSA, thus confirming a low concern for the consumer.

## 3. Materials and Methods

### 3.1. Reagents and Standards

All solvents used, including hexane, dichloromethane, acetone, and acetonitrile, were of HPLC grade and purchased from Sigma-Aldrich (Deventer, MO, USA). Potassium hydroxide (1.5 N) was obtained from Merck KGaA (Darmstadt, Germany) and prepared in water. Water was purified using a Milli-Q System (Millipore, Bedford, MA, USA).

The 610 PAH calibration standard mixture (Restek, Bellefonte, PA, USA) contained the following compounds in methylene chloride at the specified concentrations: naphthalene (Na, 1008.0 µg/mL), acenaphthylene (Ap, 1009.0 µg/mL), acenaphthene (Ac, 100.0 µg/mL), fluorene (F, 1007.2 µg/mL), phenanthrene (Pa, 500.0 µg/mL), anthracene (A, 1005.6 µg/mL), fluoranthene (Fl, 500.0 µg/mL), pyrene (P, 503.6 µg/mL), BaA (501.1 µg/mL), Ch (502.8 µg/mL), BbF (500.4 g/mL), BkF (504.0 µg/mL), BaP (500.4 µg/mL), DBahA (502.0 µg/mL), BghiP (503.6 µg/mL), and IP (504.0 µg/mL).

Silica gel 60 (particle size 63–200 µm) was obtained from Sigma-Aldrich (Deventer, MO, USA). To prevent contamination, all glassware was thoroughly washed and rinsed with acetone and distilled hexane before use.

### 3.2. Meat Samples

The meat samples consisted of pork neck from 8- or 9-month-old Yorkshire swine, sourced from a local producer. Each piece of meat weighted approximately 500 g and had a thickness of 3–5 cm.

### 3.3. Cooking Equipment

Two different cooking methods were employed: traditional grilling and an innovative cooking system called asado. The traditional grill (Figure S1) was a flat grill without a fat drainage mechanism measuring 200 cm wide, 150 cm deep, and 100 cm high. The asado (Figures S1 and S2) featured a weathering steel base and brazier, an inox 304 foot-holding cross, a grease collection channel, and a removable inox cover, measuring 66 cm wide, 85 cm deep, and 133 cm high.

### 3.4. Cooking Conditions and Sampling Plan

Cooking tests were conducted in duplicate over three separate sessions, spaced two weeks apart, following a predefined experimental plan (Table 3). The same beech wood was used as fuel for both cooking methods. For traditional grilling, embers were generated in a stainless-steel base next to the grill and then moved to the cooking area using a shovel. The distance between the coal base and the grill surface was 18 cm.

Meat and heat source temperatures were monitored every 20 min to ensure all samples reached a core temperature of 72 °C. The core temperature of the meat was measured using a wireless smart thermometer (Graphtool, San Jose, CA, USA), while flame and coal temperatures were recorded with an infrared thermometer (H-1020, Helect, London, UK).

Meat samples (16 per session), all coming from the same pig, were vacuum sealed and stored at 4 °C before cooking. Half of the samples were marinated for 12 h at 4 °C in a mixture of chives (3 g), rosemary (4 g), garlic (12 g), and Sauvignon white wine (75 mL), while the remaining samples were left untreated. After cooking, samples were wrapped in aluminum foil, labeled, and transported to the laboratory for further analysis.

Two cooking modes were tested: “fast” and “slow”. The intensity of the heating to obtain “slow” or “fast” cooking was regulated by changing the amounts of embers used. Fast cooking ranged from 100 min to 120 min on the asado grill and 70 min to 100 min on the traditional charcoal grill. Slow cooking lasted from 140 to 178 min on the asado and from 110 to 135 min on the traditional grill. Weather conditions affected cooking times, with colder days extending grilling by approximately 30 min.

### 3.5. Weight Loss and Fat Content Determination

Weight loss from cooking was measured by weighing meat samples before and after cooking, once they had cooled to room temperature. The weight after cooking was taken shortly before sample homogenization. Measurements were taken on-site with an electronic digital bench scale (AOMEX, Shenzhen, China) and confirmed in the laboratory using an analytical balance (New Classic MF, Mettler Toledo, Greifensee, Switzerland).

To determine the fat content in cooked meat, a 2 g homogenized meat sample was used for microwave-assisted extraction (MAE) with a Mars microwave extractor (CEM Corporation, Matthews, NC, USA). The extraction process involved using a 20 mL hexane/acetone mixture (3:1, *v*/*v*) at 120 °C for 20 min. After cooling, 40 mL of water was added, and the samples were frozen at −18 °C for 20 min to aid in phase separation. A 7.5 mL aliquot of the upper hexane phase was collected and concentrated using a rotary evaporator (Rotavapor, Buchi, Flawil, Switzerland) until a constant weight was achieved. The fat percentage was then calculated based on the initial sample weight and the mass extracted fat.

### 3.6. PAH Extraction and Purification

Meat samples were minced using a cooking machine (Companion XL, Moulinex, Écully, France) and stored at −18 °C until analysis. PAHs were extracted using a modified microwave-assisted saponification (MAS) and solid-phase extraction (SPE) protocol [31].

A 2.5 g minced meat sample was placed in a Teflon-lined vessel (Green Chem Plus, CEM Corporation, Matthews, NC, USA) and mixed with 10 mL hexane and 10 mL KOH (1.5 N). Saponification was performed in a Mars microwave extractor (CEM Corporation, Matthews, NC, USA) at 120 °C for 20 min. After extraction, samples were treated with 40 mL Milli-Q water and cooled to −18 °C for 20 min to enhance phase separation. Once returned to room temperature, 8 mL of the hexane phase was collected and concentrated to 200 μL using a rotary evaporator (Rotavapor, Buchi, Flawil, Switzerland) and a nitrogen flow.

The extract was purified using a 500 mg silica SPE cartridge, preconditioned with 2 mL of dichloromethane and 2 mL of hexane. PAHs were eluted with 3 mL of hexane/dichloromethane (70:30, *v*/*v*), evaporated to dryness, and reconstituted in 100 μL of acetonitrile for UHPLC analysis.

### 3.7. UHPLC Analysis

UHPLC analysis was conducted using a Shimadzu Nexera system (Shimadzu, Kyoto, Japan) equipped with two LC pumps (LC-30AD), an online degasser (DGU-20As), a column oven (CTO-30A), an autosampler (SIL-30AC), and an RF-20A-xs spectrofluorometric detector (FLD). The sampling rate was set at 10 Hz, with a 0.1 s response time.

Chromatographic conditions were adapted from [16]. The column used was a Zorbax Eclipse PAH C18 (100 mm × 2.1 mm i.d., 1.8 μm particle size, Agilent, Santa Clara, CA, USA) kept at 27 °C. The mobile phase consisted of a gradient of water and acetonitrile, running at a flow rate of 0.45 mL/min. The gradient started at 50% of acetonitrile, increased to 60% in 1.5 min, then to 75% over the next 3 min, and finally reached 100% by 8 min, maintaining this composition until 13.5 min. The injection volume was 8 μL. The excitation/emission wavelengths (λ_ex_-λ_em_) for these PAHs were set as follows: 270–390 nm for BaA and Ch, 260–430 nm for BbF, 256–410 nm for BkF and BaP, 290–410 nm for DBahA and BghiP, and 290–484 nm for IP. Quantification was performed using a seven-point calibration curve and corrected for recoveries.

### 3.8. Method Performance Assessment

Linearity was assessed by creating a seven-point calibration curve (2.5–250 µg/mL) in duplicate in acetonitrile, which corresponds to PAH8 concentrations ranging from 0.1 to 10 µg/kg of meat.

Repeatability and recovery were determined using a pan-cooked pork-neck sample with minimal contamination. Six 2.5 g portions were spiked with PAHs (2 µg/kg) and left overnight under magnetic stirring (RT-10, IKA-Werke, Staufen, Germany) to mimic analyte–matrix interactions. The remaining solvent was evaporated, and the recoveries were calculated as the percentage ratio of PAH peak areas from spiked samples (background levels subtracted) compared with calibration curve values.

## 4. Conclusions

This study evaluated the formation of PAHs during the cooking of pork using an innovative grilling system that simulates (on a small scale) traditional asado cooking, in which the meat is exposed to smoke produced by burning wood (in the presence of flames). The results obtained were compared with those obtained in parallel with a traditional flat grill (fed with beech charcoal prepared in a separate brazier) under two similar time–temperature combinations (to obtain fast and slow cooking) until reaching a core temperature of 72 °C. The use of marinade was also studied.

None of the samples analyzed surpassed the maximum limits set by European legislation for BaP (2 µg/kg) and PAH4 (12 µg/kg) in smoked meat.

No significant differences in PAH concentrations were observed between the slow and fast cooking modes, suggesting that cooking speed did not substantially affect PAH levels. Similarly, the use of marinade did not lead to statistically significant differences in PAH formation, although variations in marinade composition could yield different results.

The asado cooking method resulted in significantly higher PAH concentrations, particularly for BaP, which was approximately five times higher compared with meat cooked on the traditional flat grill. This discrepancy can be attributed to the different sources of PAHs: pyrolysis of fats during traditional grilling and the combustion of wood during asado cooking, with the latter being the predominant factor in PAH formation. Further studies are needed to better understand all parameters affecting PAH formation during meat grilling.

## Figures and Tables

**Table 1:** Heating source temperatures, cooking durations, and climatic conditions from three experimental sessions using flat and asado grills.

Cooking Session	Grill Type	Cooking Mode	Heating Source		Climatic Conditions
			Temperature Range (°C)	Time Range (min)	
I	flat	slow	246–367	135	6.3–10.4 °C; RH: 93%; cloudy day
		fast	464–544	100	
	asado	slow	249–350	178	
		fast	475–553	120	
II	flat	slow	292–342	110	7.6–16.2 °C; RH: 36%; sunny day
		fast	423–523	80	
	asado	slow	282–352	140	
		fast	482–531	95	
III	flat	slow	281–330	110	7.8–15.9 °C; RH: 38%; cloudy day
		fast	454–522	70	
	asado	slow	283–368	160	
		fast	447–533	110	

RH: relative humidity.

**Table 2:** Average (*n* = 6) data on weight loss and fat content in meat cooked using a traditional flat grill and an asado grill under different conditions (fast and slow cooking, with and without marinade).

	Traditional Flat Grill				Asado Grill			
Cooking mode	Fast		Slow		Fast		Slow	
Marinade	yes	no	yes	no	yes	no	yes	no
Weight loss (%)	29.2	37.2	34.8	34.4	31.6	35.8	29.0	32.2
SD (%)	6.7	7.6	5.7	4.1	4.8	6.7	5.1	6.0
RSD (%)	23.0	20.4	16.4	12.0	15.2	18.7	17.6	18.7
Fat content (%)	24.9	22.0	25.9	26.6	24.1	21.5	24.0	23.4
SD (%)	3.6	3.8	11.1	4.7	8.1	5.9	4.0	7.1
RSD (%)	14.4	17.3	43.0	17.6	33.4	27.3	16.6	30.4

SD: standard deviation; RSD: relative standard deviation.

**Table 3:** Experimental plan followed during cooking tests.

Grill Type	Asado Grill				Traditional Flat Grill			
Heating source	flame from beech wood				charcoal from beech wood			
Fat dripping	limited				yes			
Cooking speed (to reach 72 °C at the core)	slow		fast		slow		fast	
Marinating	no	yes	no	yes	no	yes	no	yes
Cooking session	3	3	3	3	3	3	3	3
Replicate number for each cooking session	2	2	2	2	2	2	2	2
Total pieces of meat cooked in the 3 sessions	24				24			

## Data Availability

Data are contained within the article.

## Supplementary Materials

The following supporting information can be downloaded at: https://www.mdpi.com/article/10.3390/molecules30091886/s1. Figure S1. Asado and traditional flat grill. The left image illustrates pork neck being cooked using an asado grill, where the meat is positioned vertically near an open flame from burning beech wood. The right image depicts the traditional flat grill setup, where the pork neck rests directly on a metal grate over glowing beech charcoal embers; Figure S2. Frontal (left) and lateral (right) views of asado grilling; Table S1. Summary of analytical performance parameters for the determination of PAHs, including regression equations, coefficients of determination (R^2^), recoveries (*n* = 6, spiked concentration of 2 µg/kg), limits of detection (LOD), and limits of quantification (LOQ); Table S2. PAH concentrations, expressed as µg/kg, in grilled food samples under different cooking conditions.

## Abbreviations

The following abbreviations are used in this manuscript:

BaA	Benzo[a]anthracene
BaP	Benzo[a]pyrene
BbF	Benzo[b]fluoranthene
BghiP	Benzo[g,h,i]perylene
BkF	Benzo[k]fluoranthene
Ch	Chrysene
DBahA	Dibenzo[a,h]anthracene
EFSA	European Food Safety Authority
FLD	Fluorometric detector
IP	Indeno [1,2,3-cd]pyrene
MAE	Microwave-assisted extraction
MAS	Microwave-assisted saponification
PAH	Polycyclic aromatic hydrocarbons
PAH4	Sum of chrysene, benzo[a]anthracene, benzo[a]pyrene, and benzo[b]fluoranthene
PAH8	Sum of PAH4 and benzo[k]fluoranthene, dibenzo[a,h]anthracene, benzo[g,h,i]perylene, and indeno [1,2,3-cd]pyrene
SPE	Solid-phase extraction
UHPLC	Ultra-high performance liquid chromatography
