# Supplementary material for: Evaluation of Polycyclic Aromatic Hydrocarbons (PAHs) in Pork Meat Cooked with Two Different Methods
Source: Molecules. 2025 Apr 23;30(9):1886. doi: 10.3390/molecules30091886 (PMC12073552; doi:10.3390/molecules30091886)
Supplement: Supplementary file 1 [file molecules-30-01886-s001.zip › molecules-3590250-supplementary.pdf]

Article

Evaluation of polycyclic aromatic hydrocarbons (PAH) in pork meat cooked with two different methods

Chiara Conchione, Silvia Socal, Laura Barp and Sabrina Moret*

[1] Department of Agri-Food, Environmental and Animal Sciences, University of Udine, Udine, Italy; chiara.conchione@uniud.it (C.C.), laura.barp@uniud.it (L.B.)

* Correspondence: sabrina.moret@uniud.it

Supplementary Materials

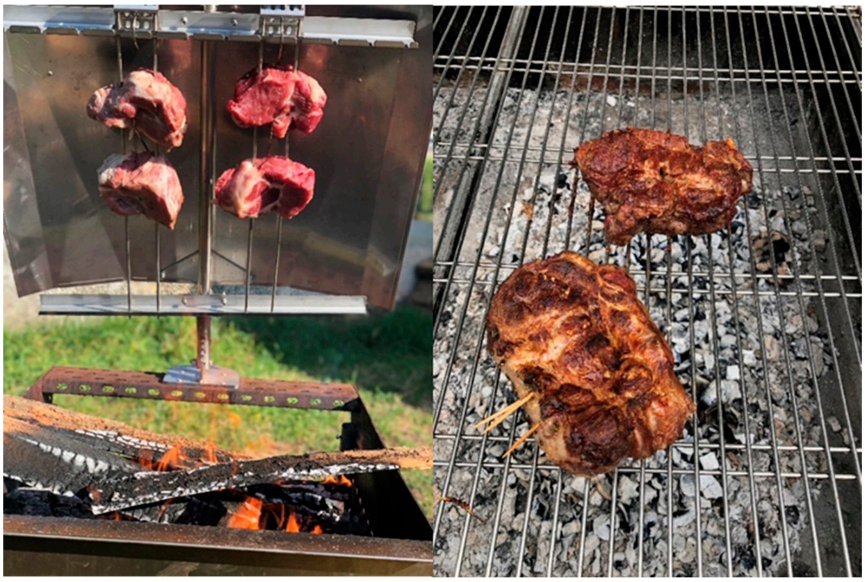

Figure S1. Asado and traditional flat grill. The left image illustrates pork neck being cooked using an asado grill, where the meat is positioned vertically near an open flame from burning beech wood. The right image depicts the traditional flat grill setup, where the pork neck rests directly on a metal grate over glowing beech charcoal embers.

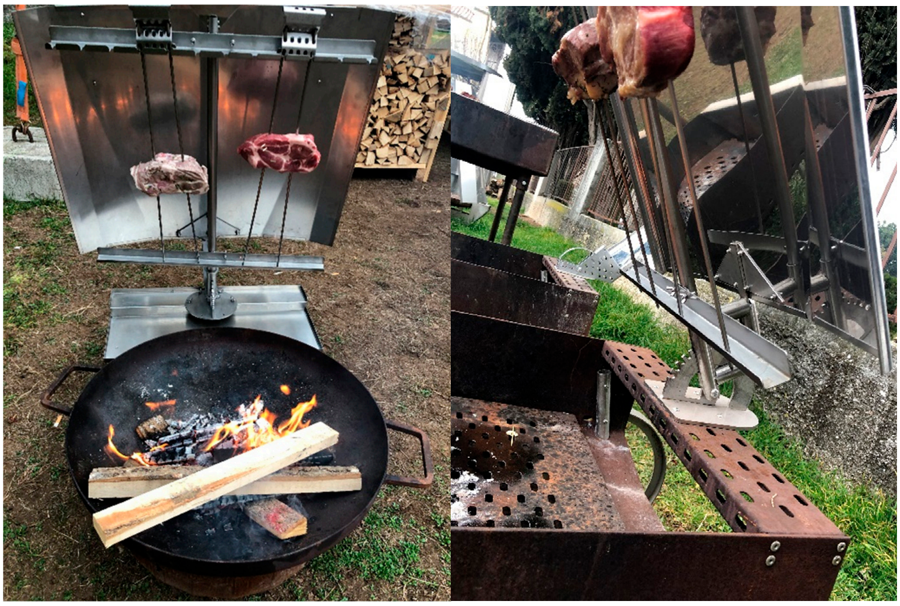

Figure S2. Frontal (left) and lateral (right) views of asado grilling.

Table S1. Summary of analytical performance parameters for the determination of PAHs, including regression equations, coefficients of determination (R^2), recoveries (n=6, spiked concentration of 2 μg/kg), limits of detection (LOD), and limits of quantification (LOQ).

PAH	Equation of regression line	R^2	Recovery (%)	RSD (%)	LOD (μg/kg)	LOQ (μg/kg)
BaA	y = 2e+06x - 161250	0.9998	79.5	5.9	0.01	0.05
Ch	y = 1e+06x - 118941	0.9995	97.2	5.5	0.02	0.07
BbF	y = 777043x - 64776	0.9998	94.0	9.2	0.02	0.08
BkF	y = 2e+06x - 106786	0.9991	91.7	6.0	0.01	0.03
BaP	y = 1e+06x + 80008	0.9899	98.1	7.3	0.02	0.06
DBahA	y = 1e+06x - 128111	0.9994	93.6	9.7	0.04	0.12
BghiP	y = 1e+06x - 119298	0.9992	93.2	5.2	0.02	0.06
IP	y = 146715x - 16908	0.9990	89.1	10.4	0.04	0.12

Table S2. PAH concentrations, expressed as μg/kg, in grilled food samples under different cooking conditions.

Cooking session	Grilling type	Cooking velocity	Marinade	BaA	Ch	BbF	BkF	BaP	DBahA	BghiP	IP	PAH4	PAH8
1st (A)	Flat	fast	yes	0.90 ± 0.05	0.81 ± 0.22	0.25 ± 0.05	0.23 ± 0.03	0.56 ± 0.10	0.24 ± 0.06	0.20 ± 0.06	0.28 ± 0.07	2.52 ± 0.42	3.49 ± 0.65
			no	0.64 ± 0.08	0.64 ± 0.06	0.21 ± 0.06	0.16 ± 0.04	0.24 ± 0.01	0.31 ± 0.09	0.14 ± 0.01	0.17 ± 0.01	1.73 ± 0.22	2.51 ± 0.37
		slow	yes	0.50 ± 0.06	0.79 ± 0.25	0.46 ± 0.14	0.16 ± 0.03	0.23 ± 0.02	0.30 ± 0.07	0.15 ± 0.01	0.62 ± 0.34	1.98 ± 0.47	3.22 ± 0.92
			no	1.11 ± 0.09	1.31 ± 0.09	4.51 ± 0.35	0.10 ± 0.01	0.33 ± 0.13	0.40 ± 0.15	0.15 ± 0.01	1.26 ± 0.02	7.26 ± 0.66	9.17 ± 0.84
	Asado	fast	yes	0.42 ± 0.15	0.52 ± 0.20	0.66 ± 0.43	0.12 ± 0.03	0.37 ± 0.24	0.20 ± 0.02	0.24 ± 0.11	0.33 ± 0.08	1.97 ± 1.02	2.86 ± 1.26
			no	0.97 ± 0.06	1.25 ± 0.05	0.72 ± 0.18	0.32 ± 0.02	1.00 ± 0.01	0.37 ± 0.01	0.25 ± 0.01	0.36 ± 0.01	3.93 ± 0.31	5.23 ± 0.34
		slow	yes	1.24 ± 0.03	1.33 ± 0.20	1.15 ± 0.01	0.20 ± 0.02	0.93 ± 0.20	0.22 ± 0.04	0.39 ± 0.26	0.83 ± 0.05	4.65 ± 0.44	6.28 ± 0.80
			no	0.86 ± 0.03	1.22 ± 0.06	0.84 ± 0.03	0.25 ± 0.01	0.88 ± 0.02	0.33 ± 0.02	0.16 ± 0.01	0.37 ± 0.06	3.80 ± 0.14	4.91 ± 0.23
2nd (B)	Flat	fast	yes	0.52 ± 0.10	0.60 ± 0.04	0.19 ± 0.01	0.18 ± 0.02	0.25 ± 0.06	0.18 ± 0.02	0.14 ± 0.01	0.13 ± 0.01	1.55 ± 0.21	2.19 ± 0.26
			no	0.29 ± 0.06	0.45 ± 0.13	0.15 ± 0.02	0.14 ± 0.03	0.21 ± 0.07	0.21 ± 0.01	0.16 ± 0.01	0.18 ± 0.04	1.10 ± 0.28	1.80 ± 0.36
		slow	yes	0.31 ± 0.06	0.31 ± 0.01	0.19 ± 0.02	0.10 ± 0.01	0.10 ± 0.04	0.15 ± 0.01	0.17 ± 0.01	0.32 ± 0.07	0.91 ± 0.13	1.65 ± 0.21
			no	0.29 ± 0.11	0.32 ± 0.11	0.14 ± 0.02	0.10 ± 0.03	<LOQ	0.16 ± 0.01	0.16 ± 0.03	0.22 ± 0.08	0.76 ± 0.25	1.40 ± 0.40
	Asado	fast	yes	1.31 ± 0.19	1.84 *	n.q.	0.34 *	1.43 *	0.22 ± 0.01	0.73 ± 0.03	0.15 ± 0.01	4.59 ± 0.19	6.02 ± 0.24
			no	0.47 ± 0.02	1.40 ± 0.18	n.q.	0.16 *	2.22 *	0.37 ± 0.13	1.06 ± 0.06	0.71 ± 0.38	4.09 ± 0.20	6.39 ± 0.77
		slow	yes	0.58 ± 0.47	1.77 ± 0.19	n.q.	0.32 ± 0.01	1.32 *	0.13 ± 0.13	0.41 ± 0.41	n.q.	3.68 ± 0.66	4.54 ± 1.20
			no	0.69 ± 0.12	1.20 ± 0.02	1.06 ± 0.44	0.24 ± 0.03	1.10 ± 0.01	<LOQ	0.20 ± 0.20	n.q.	4.04 ± 0.59	4.48 ± 0.82
3rd (C)	Flat	fast	yes	0.25 ± 0.03	0.18 ± 0.01	0.19 ± 0.01	0.08 ± 0.01	0.11 ± 0.06	0.15 ± 0.01	0.17 ± 0.01	0.15 ± 0.01	0.73 ± 0.10	1.28 ± 0.13
			no	0.24 ± 0.08	0.18 ± 0.02	0.15 ± 0.01	0.08 ± 0.02	0.12 ± 0.15	0.17 ± 0.03	0.14 ± 0.01	0.16 ± 0.02	0.69 ± 0.25	1.24 ± 0.31
		slow	yes	0.22 ± 0.09	0.35 ± 0.19	0.18 ± 0.05	0.07 ± 0.01	<LOQ	0.17 ± 0.03	0.87 ± 0.67	0.25 ± 0.08	0.75 ± 0.32	2.11 ± 1.10
			no	0.13 ± 0.01	0.22 ± 0.06	0.17 ± 0.01	0.10 ± 0.02	0.07 ± 0.07	0.15 ± 0.01	0.16 ± 0.02	0.31 ± 0.18	0.59 ± 0.14	1.30 ± 0.36
	Asado	fast	yes	1.12 ± 0.08	1.76 ± 0.05	1.11 ± 0.25	0.51 ± 0.23	1.38 ± 0.11	0.24 ± 0.03	0.57 ± 0.16	1.16 ± 0.24	5.36 ± 0.48	7.84 ± 1.13
			no	0.73 ± 0.27	1.30 ± 0.77	0.75 ± 0.18	0.36 ± 0.09	1.52 ± 0.01	0.26 ± 0.07	0.68 ± 0.27	1.37 ± 0.33	4.29 ± 1.21	6.96 ± 1.98
		slow	yes	0.42 ± 0.24	1.24 ± 0.01	1.05 ± 0.53	0.28 ± 0.08	1.34 ± 0.38	0.25 ± 0.05	0.60 ± 0.27	1.15 ± 0.56	4.06 ± 1.15	6.33 ± 2.12
			no	0.50 ± 0.19	0.77 ± 0.35	0.24 ± 0.05	0.60 ± 0.51	1.07 ± 0.85	0.18 ± 0.03	0.41 ± 0.23	0.75 ± 0.50	2.58 ± 1.44	4.53 ± 2.70

*: single sample (no SD); n.q.: not quantifiable; <LOQ: below the limit of quantification.
